# Indications for magnetic resonance imaging of the female pelvis at a referral center for cancer

**DOI:** 10.1590/0100-3984.2017.50.1e1

**Published:** 2017

**Authors:** Patrícia Prando Cardia

**Affiliations:** 1 Médica Radiologista do Centro Radiológico Campinas/Hospital Vera Cruz e do Hospital e Maternidade Celso Pierro da Pontifícia Universidade Católica de Campinas (PUC Campinas), Campinas, SP, Brasil. E-mail: pprando@diagnostiqueimagens.com.br.

Guidelines that review the criteria and technical parameters for indicating and conducting diagnostic imaging can optimize radiology care. In an article published in this issue of **Radiologia Brasileira**, the criteria established by the American College of Radiology for conducting magnetic resonance imaging (MRI) examinations of the female pelvis were compared with those employed at a national referral center for cancer in Brazil^(1)^. The results were compatible with those of the renowned American institution and show that MRI was correctly indicated in most (76.7%) of the patients. The authors found that the main indications were as follows: tumor recurrence after resection (25.9%); detection and staging of malignancies (23.3%); and evaluation of pain or pelvic mass (17.1%).

There is a well-defined role for MRI in the study of the female pelvis after the initial ultrasound evaluation, as has been amply demonstrated in the literature^(2,3)^. When the examination is indicated in the appropriate clinical context, the inherent characteristics of the method (related to its multiplanar capacity, the possibility of tissue differentiations of the pelvic organs, and the absence of ionizing radiation) outweigh its disadvantages (longer examination time, possible motion artifacts, lower availability, and higher costs) when compared with computer tomography.

As observed by the authors of the cited study^(1)^, one of the main indications for MRI of the female pelvis is for the diagnosis or oncological follow-up of primary gynecological lesions. In relation to primary uterine lesions, diffusion-weighted imaging facilitates the identification of tumors in the uterine body and cervix^(4)^. The use of diffusion-weighted imaging also facilitates the restaging of such tumors after chemotherapy and radiotherapy, as well as allowing the identification of tumor remnants and recurrence. These diagnostic processes tend to be challenging, since actinic and fibrotic alterations, which are typical of these treatments, alter the morphology of the pelvic organs^(5,6)^.

Still within the field of oncology, MRI is frequently used for the evaluation of adnexal lesions that are considered "indeterminate" in the ultrasound study^(2,7)^. The interpretation of an MRI of the female pelvis begins with the characterization of the origin of the pelvic lesion as ovarian or extraovarian. Specifically, when the origin is defined as ovarian, the characterization of the complex lesions is followed by an assessment of the solid components, papillary projections, intralesional septations, and vascularization. Criteria have recently been proposed for the scoring of these findings, which together with the information obtained in the diffusionweighted sequences and in the dynamic study after administration of intravenous contrast medium, allow an assessment of the malignancy criteria^(8)^.

Outside the cancer centers, MRI is usually requested to evaluate benign diseases of the female pelvis, as stated by the American College of Radiology^(9)^. The study of the vagina, the characterization of urethral and periuretheral lesions, the evaluation of Müllerian malformations, and especially the study of myomatosis, adenomyosis, and pelvic endometriosis are the main indications for MRI of the female pelvis in daily practice at such diagnostic centers^(10-13)^.

The dynamic evaluation of the pelvic floor, through MRI defecography, has been replacing the study of traditional defecography, and proctologists increasingly value the MRI findings, since the information obtained goes far beyond the anatomical evaluation of the pelvic organs, with characterization of rectocele, enterocele, anorectal invagination, and anismus^(14)^.

In the case of MRI in pregnant patients, the safety of the fetus after the first trimester supports the indication for the test in the evaluation of pregnancy-related complications and intercurrent diseases during pregnancy^(15,16)^.

Considering the aspects presented, it can be concluded that the indications for performing MRI of the female pelvis cover a broad spectrum of benign and malignant conditions, in well-established clinical contexts, both nationally and internationally. In Brazil, the validation of international guidelines proposed by recognized radiological entities underscores the value of this information. It also allows greater dissemination, since the radiologist also has the role of guiding the medical community regarding the appropriate indications for the various imaging methods.
